# Isolated left ventricular non-compaction cardiomyopathy complicated by acute ischemic stroke: A rare case repor

**DOI:** 10.1016/j.amsu.2022.104543

**Published:** 2022-09-01

**Authors:** Said Abdirahman Ahmed, Mesut Karataş, Lütfi Öcal, Mohamed Sheikh Hassan, Mohmed Abdullahi Mohamud, Mohamed Omar Hassan, Abdirahman Mohamed Hassan Dirie, Mohamud Mire Waberi, Abdijalil Abdullahi Ali

**Affiliations:** a Cardiology Department at Mogadishu Somali-Turkish Training and Research Hospital, Mogadishu, Somalia; b Neurology Department at Mogadishu Somali-Turkish Training and Research Hospital, Mogadishu, Somalia; c Pulmonolgy Department at Mogadishu Somali-Turkish Training and Research Hospital, Mogadishu, Somalia; d Cardiovascular Surgery Department at Mogadishu Somali-Turkish Training and Research Hospital, Mogadishu, Somalia

**Keywords:** Non-compaction cardiomyopathy, Non-sustained ventricular tachycardia, Holter monitoring, Cardiomyopathy, Electrocardiography

## Abstract

**Introduction:**

and importance: Isolated left ventricular noncompaction cardiomyopathy (LVNC), uncommon type of primary hereditary cardiomyopathy. It is a spongy morphological appearance of the myocardium that occurs largely in the LV.

**Case presentation:**

We discuss here a case of 19 years old female with no known past medical history who present with Shortness of breath (SOB) and left sided weakness following delivery.

Bedside Echocardiography demonstrated Left ventricular trabiculation with reduced ejection fraction. While brain Computed tomography showed acute ischemic stroke primly due to non-compaction cardiomyopathy as the embolic. Patient was discharged after successfully managed.

**Clinical discussion:**

Left ventricular non-compaction cardiomyopathy (LVNC) is characterized by progressive ventricular trabeculation and deep intratrabecular recesses caused by the functional arrest of myocardial maturation, which is a rare case of congenital cardiomyopathy. Our patient had isolated non-compaction cardiomyopathy of the type that was complicated by an acute ischemic stroke and was treated accordingly.

**Conclusion:**

It is usually associated with congenital heart disease, but isolated left ventricular non-compaction cardiomyopathy is very uncommon.

## Highlights

• Adults with left ventricular noncompaction are at risk for significant consequences such stroke, HF, arrhythmia, and mortality.
• No evidence-based guidelines for preventing thromboembolic events in isolated left ventricular noncompaction.
• Transthoracic echocardiography remains the most commonly used method for LVNC diagnosis.

## 1 Introduction

Left ventricular noncompaction (LVNC), also known as “spongy myocardium,” is a rare abnormality of the left ventricular (LV) wall caused by a failure of the myocardium's normal first-trimester compaction process, resulting in the formation of two layers of myocardium: the compacted layer and the non-compacted layer [1].

It is usually associatedwith arrhythmias, congenital heart disease, including septal defects right heart obstructive abnormalities including pulmonic stenosis and Ebstein's anomaly, and hypoplastic left heart syndrome [2].

Although in observational studies, LVNC has been discovered in 0.01–0.26% of all individuals referred to an echocardiography lab, the prevalence of isolated LVNC in adults is still unknown [3].

We present here a Isolated Left Ventricular Non-Compaction cardiomyopathy Complicated By Acute Embolic Stroke.

## 2 Case presentation

A 19-year-old female came to the emergency via ambulance due to shortness of breath (SOB), diaphoresis, and left-sided weakness after an uncomplicated vaginal delivery. She had no chronic diseases as well as a known family history. In an emergency, the vitals were as follows: Spo2 82, glucose 97, Bp 120/60, and a pulse of 150bpm.

On cardiac examination; there was tachycardia, normal heart sound, with no added sounds.

**Fig. 1:**
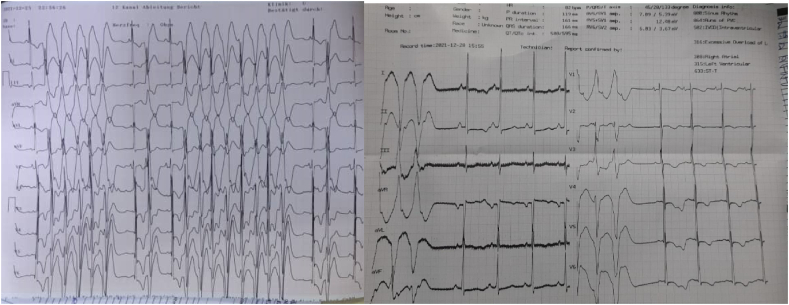
**anel A shows** non-sustained ventricular tachycardia with heart rate of 180pbm**. Panel B** after amiodrone ECG shows triple premature ventricular contraction.

**Fig. 2:**
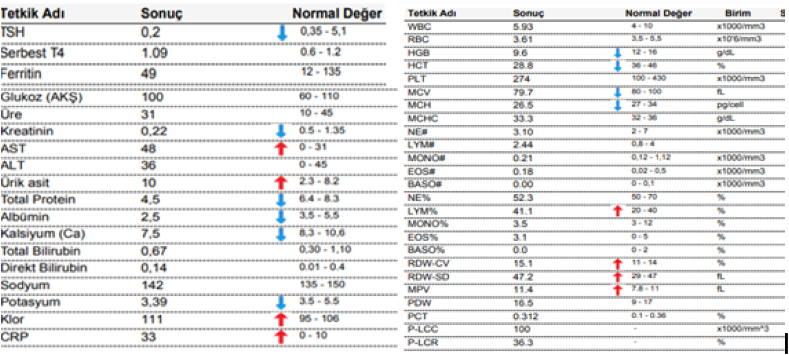
Basic lab test with electrolytes being in normal as well as thyroid and full blood count.

**Fig. 3:**
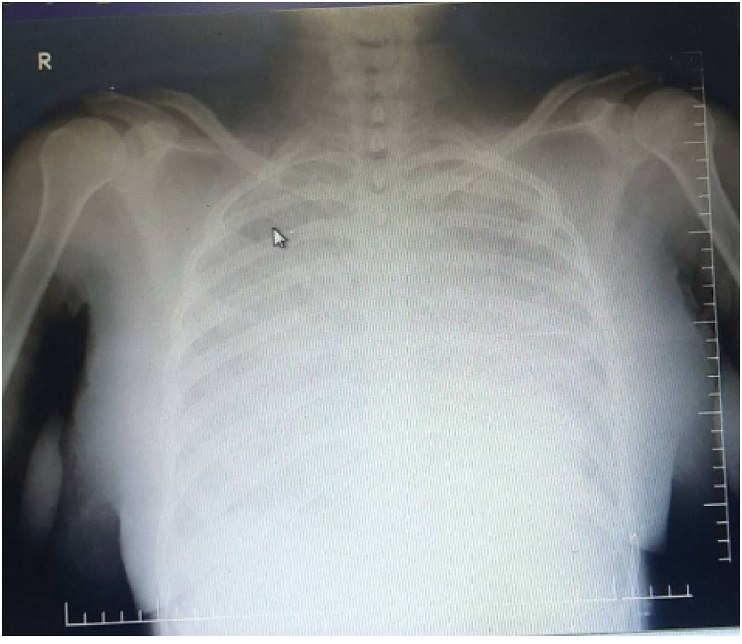
**Chest x ray** shows Wight opacification consistent with pulmonary edema.

On lung auscultation, there were reduced breath sounds. Other systems were unremarkable except for left-sided weakness and grade 2 lower limb edema. 12 electrocardiography (ECG) showed non-sustained ventricular tachycardia and a pulse of 180bpm figure (1). To rule out aggravating factors of non-sustained ventricular tachycardia, basic laboratory tests such as a full blood count and thyroid function tests were performed, yielding the following results in figure (2) while a chest x-ray revealed pulmonary edema as shown in figure (3)**.**

Bedside echocardiography was done to exclude postpartum cardiomyopathy as the cause of her symptoms.

**Fig. 4:**
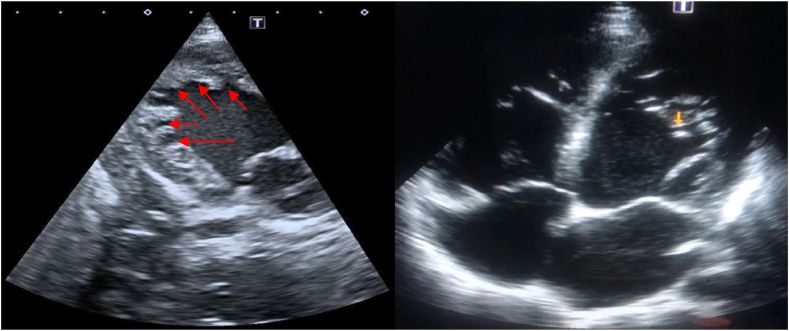
**Panel A** Para-sternal short axis views. **Panel B** apical 4 champers with left ventricular shows trabiculaion.

The echocardiography showed trabeculation of the left ventricles, and heart failure reduced ejection fraction (35–40%), as shown in figure (4).

**Fig. 5:**
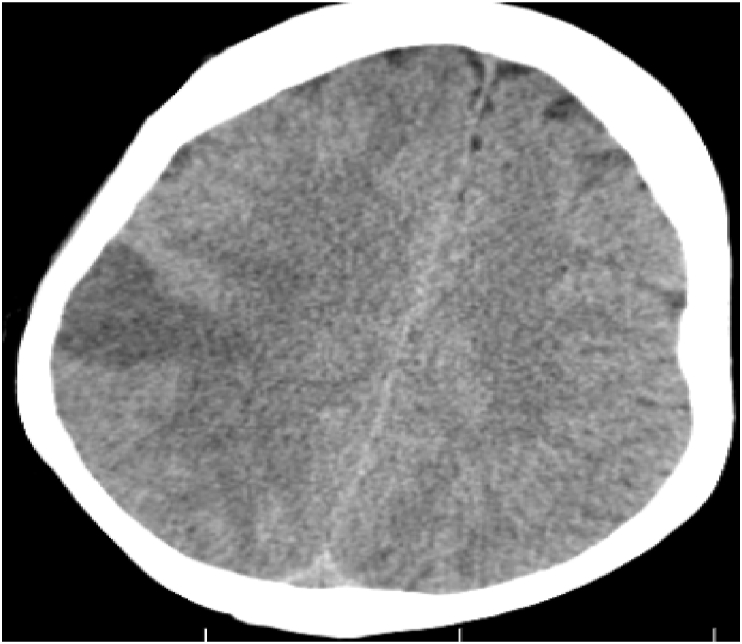
**Brain CT** shows right parietal infract.

The patient was transferred to the Intensive care unit (ICU) and the amiodarone loading dose, potassium replacement, aspirin (empirically), and furosemide infusion with continuous positive airway pressure (CPAP) were started. On day two after stabilization of the patient, a brain CT scan showed an acute ischemic infarct figure (5). The patient was transferred to an inpatient due to clinical improvement and resolution of pulmonary edema with the addition of bisoprolol 5mg 1x1 and enalapril 2.5mg 2x1. The patient was discharged with aspirin, bisoprolol 5mg/1x1, and enalapril 2.5mg 2x1, and dietary advice was given. On follow-up week two, the patient was healthy and titration of heart failure management was made.

## 3 Discussion

LVNC) is a rare type of primary hereditary cardiomyopathy that happens when the ventricular myocardium stops developing during embryogenesis [4]. It is a spongy morphological appearance of the myocardium that occurs mainly in the LV. These irregular trabeculations are often more visible in the apical and midlateral–inferior parts of the LV [5].

In one large group of adult patients, the prevalence of LVNC was 0.014%. The majority of people with LVNC remain asymptomatic; roughly two-thirds of them develop heart failure as a result of ventricular dysfunction, although embolic events, arrhythmias, and sudden cardiac death are less prevalent [6].

No sustained ventricular tachycardia (NSVT) is described as 3 (occasionally 5) or more successive beats occurring below the atrioventricular node with an RR interval of 600 ms (100 beats/min) and lasting 30 seconds [7]. Given this cardiomyopathy's particular arrhythmogenic tendency, the same hemodynamic load can also cause an arrhythmic risk during pregnancy. Likewise, our case was asymptomatic in her life and presented with left ventricular dysfunction and an acute stroke following delivery. Patients with LVNC frequently develop left ventricular systolic dysfunction [8]. The ejection fraction (EF) in this case was 35–40%.

Stroke/Embolic in patients with LVHT/NC is not always cardioembolic; it can also have an atherosclerotic origin. In individuals with LVHT/NC without AF or left ventricular systolic failure, cardioembolic strokes or emboli are infrequent [9].

Due to the rarity of the disease [10], there are currently no evidence-based guidelines for preventing thromboembolic events in isolated left ventricular noncompaction.

Echocardiographic, cardiac magnetic resonance (CMR), and computed tomography (CT) imaging criteria can be used to make the diagnosis of this cardiomyopathy. However, none of these imaging methods has been standardized to provide a definitive diagnosis [11].

Because it is widely available, easily interpreted, and inexpensive, transthoracic echocardiography remains the most commonly used method for LVNC diagnosis [12].

The ratio of the non-compacted layer to the compacted layer thickness (T/C) is one of the most often used criteria for diagnosis, with a ratio of >2 at end-diastole being regarded as diagnostic [13]. Due to the lack of cardiac MRI in our country, echocardiography was used as a diagnosis.

In comparison to patients with DCM, those with LVNC had comparable risks of cardiovascular mortality, all-cause mortality, thromboembolic consequences, and ventricular arrhythmias [14]. This case has been reported in line with the SCARE criteria [15].

## 4 Conclusion

Adults with left ventricular noncompaction are at risk for significant consequences such HF, stroke, arrhythmia, and mortality.

Anticoagulation is not advised as a preventative measure because of the risk of thromboembolism.

## Ethical approval

According to our hospital rule, Ethical approval is only required in articles but not case reports.

## Sources of funding

There is no funding source for this study.

## Author contribution

All authors contributed toward writing, analysis, drafting, and revising the paper and they gave final approval of the version to be published, and agree to be accountable for all aspects of the work.

## Conflicts of interest

I declare that there is no competing interest related to the study, authors, other individuals, or organizations.

## Registration of research studies

1 Name of the registry: Not applicable
2 Unique Identifying number or registration ID: Not applicable
3 Hyperlink to your specific registration (must be publicly accessible and will be checked): Not applicable

## Guarantor

Said Abdirahman Ahmed.

## Consent for publication

Written informed consent was obtained from the patient for publication of this case report and accompanying images. A copy of the written consent is available for review by the Editor-in-Chief of this journal on request.

## Availability of data and materials

N/A.

## Provenance and peer review

Not commissioned, externally peer-reviewed.
